# The iModulon framework: how x-AI reveals microbial regulatory logic

**DOI:** 10.1128/msystems.00228-26

**Published:** 2026-08-12

**Authors:** Kangsan Kim, Edward Alexander Catoiu, Yongjae Lee, Dukwon Lee, Chaewon Lee, Jiwon Lee, Jongoh Shin, Bernhard Palsson, Byung-Kwan Cho

**Affiliations:** 1 Graduate School of Engineering Biology, Korea Advanced Institute of Science and Technology 34968, Daejeon, Republic of Korea; 2 KI for the BioInnovation, Korea Advanced Institute of Science and Technology 34968, Daejeon, Republic of Korea; 3 Department of Bioengineering, University of California San Diego 207027, La Jolla, California, USA; 4 Department of Biological Sciences, Chonnam National University 34931, Gwangju, Republic of Korea; 5 Institute of Synthetic Biology for Carbon Neutralization, Chonnam National University 34931, Gwangju, Republic of Korea; Rice University, Houston, Texas, USA; Pacific Northwest National Laboratory, Richland, Washington, USA

**Keywords:** transcriptomics, systems biology, AI

## Abstract

The accelerating deposition of RNAseq data over the past decade has motivated the development of advanced transcriptomic data analytics that can operate on a large number of samples. One successful approach is to apply independent component analysis (ICA) to large prokaryotic transcriptomic compendia to decompose them into independently modulated gene sets, called iModulons. Here, we review the data science principles underlying ICA-based transcriptome decomposition, computational workflows that support its routine application, and iModulonDB infrastructure that hosts and disseminates the resulting decompositions. We present iModulonDB 3.0 that contains 53 species and 71 ICA decompositions across 33,062 RNA-seq samples, with several well-sampled species (e.g., *Escherichia coli*, *Bacillus subtilis*, *Staphylococcus aureus, Pseudomonas aeruginosa*) represented by more than one compendium. With 71 standardized decompositions, we demonstrate systematic cross-species comparison of species-specific iModulon structures. This comparison identifies a shared “regulatory toolkit” of 13 modules conserved across distantly related bacteria, alongside a long tail of lineage-specific programs. We assess the design principles and limitations governing iModulon reconstruction and computation. Together, these advances position the iModulon framework as an accessible, community-driven approach for reading accumulating public transcriptomes as reusable regulatory programs, enabling biological discovery and module-level design in synthetic biology.

## INTRODUCTION

**Fig 1:**
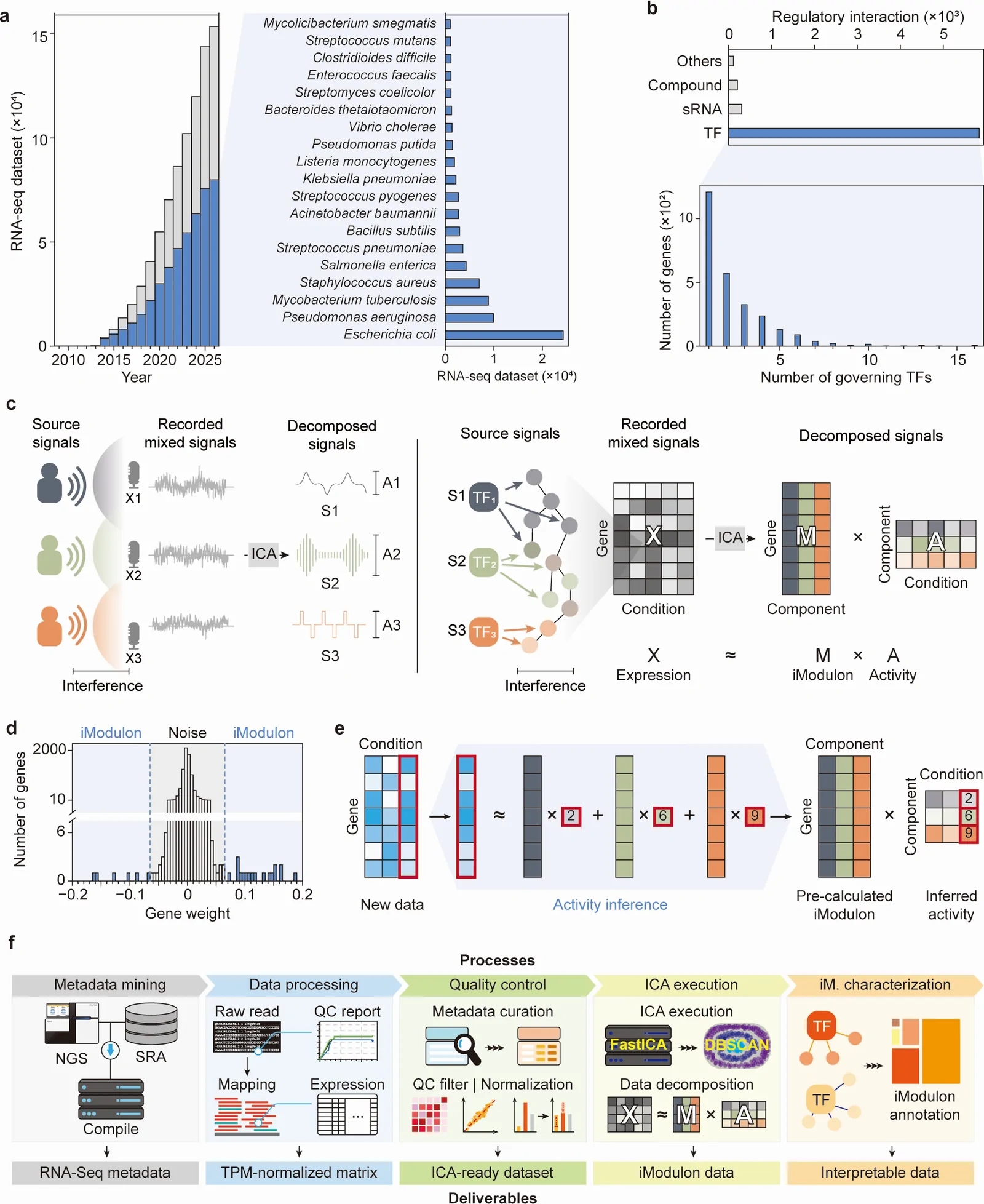
Prokaryotic transcriptome analysis through independent component analysis. (**a**) Overview of the over 150,000 publicly available bacterial transcriptomic data sets reported in the NCBI sequence read archive. Nineteen microbial species with over 1,000 RNA-seq data sets. (**b**) Characteristics of the regulatory architecture of *E. coli*. (**c**) Schematic of independent component analysis (ICA) on large-scale transcriptomic data set. ICA extracts statistically independent source signals from observed mixed signals under the assumption that the underlying sources are mutually independent, providing both the extracted source signals and their activities across the observations. In the classic acoustic analogy (left), microphones capture the mixed voices of multiple speakers at varying volumes. By maximizing statistical independence, ICA mathematically deconvolutes these mixed recordings *X*(*n*) into the original independent source voices *S*(*n*) and their relative intensities *A*(*n*). Similarly, for transcriptomics (right), the same principle can be applied to prokaryotic transcriptomic compendia by assuming each transcriptome as a mixture of independent regulatory activities. Applying ICA to a transcriptomic compendium decomposes the data into independent regulatory signals represented by the **M** matrix (gene composition of iModulons) and their condition-specific activities represented by the **A** matrix (iModulon activities). (**d**) Schematic representation of the determination of gene composition of iModulon. The histogram shows the gene weightings in the column of **M** that represents an iModulon. Weightings that are statistically significant determine the set of independently modulated genes. (**e**) Exploiting previously calculated iModulons for analyzing the iModulon composition of newly generated transcriptomic data sets using an existing iModulon structure. (**f**) Schematic representation of the iModulonMiner pipeline.

A transcriptome can be regarded as the sum of all regulatory activities under the investigated condition. It enables us to reveal how regulatory systems and biological functions are organized and modulated (1). Advances in next-generation sequencing (NGS) have driven rapid accumulation of gene expression profiles across diverse organisms, including microbes. Since 2009, more than 150,000 bacterial RNA-seq data sets have been generated and made publicly available, as cataloged in the NCBI sequence read archive (SRA) (Fig. 1a) (2). The extensive number of available transcriptomes reflects the depth of condition sampling within individual species. As of April 2026, 19 bacterial species have each accumulated more than 1,000 publicly available RNA-seq data sets (Fig. 1a).

Beyond differential expression analysis across a handful of conditions, large per-species transcriptome compendia now allow machine-learning approaches to characterize global regulatory structure (3). Among these, independent component analysis (ICA) stands out for recovering architectures that align closely with known transcription factor (TF)–DNA interaction networks (4). For an experimental biologist, the practical appeal of this decomposition is that it converts a transcriptome from a list of thousands of individually varying genes into a smaller set of dozens of co-regulated modules, each corresponding to a recognizable cellular function. This reframing allows one to ask module-level questions—which functions are switched on under an experimental condition, when a program is deployed, and how its activity tracks with phenotype—and offers a route to place genes of unknown function into physiological context, refine the boundaries of biological units, and compare regulatory states across strains and conditions. We return to these applications, including emerging uses in module-level strain design, in “Outlook” below; here, we describe the data science framework that generates iModulon structures for prokaryotic transcriptomes.

## FROM ICA TO iModulons

ICA was originally developed to separate mixed audio signals under the assumption that each source signal is statistically independent of the others (Fig. 1c) (5). The same principle applies remarkably well to regulation of gene expression at the genome scale, where the independence of regulatory signals is a strong empirical feature (1, 6). In prokaryotes, most genetic regulation occurs at the level of transcription initiation, mediated primarily by transcription factors (TFs). It has been argued that this approach to transcriptional regulation minimizes unnecessary expenditure of cellular resources in the downstream steps of the central dogma (7). In addition, the regulons of individual TFs—the sets of target genes characterized experimentally through ChIP-seq, TF knockouts, and related approaches—have limited overlap with other regulons. In *E. coli*, for example, around 90% of the 6460 regulatory interactions documented in RegulonDB are mediated by TFs, and about 67% of TF-regulated genes are known to be regulated by only one or two TFs (Fig. 1b) (8). Such regulatory architecture provides a strong rationale for applying ICA to prokaryotic transcriptomes, as it is broadly consistent with ICA’s assumption of partially independent source signals.

In a comprehensive benchmarking analysis utilizing transcriptomics data from prokaryotic and eukaryotic data sets, ICA outperformed 42 other module detection algorithms, including clustering, biclustering, and decomposition methods, in recovering known biological regulons (4). In particular, among decomposition approaches, such as principal component analysis (PCA) and non-negative matrix factorization (NMF), ICA was superior in producing reproducible and generalizable components—that is, co-regulated gene sets (9). Furthermore, ICA achieved significantly higher true-positive rates, demonstrating superior recall in mapping the extracted components to known biological systems (10). These differences are attributed to the inherent constraints of each algorithm. For example, PCA enforces orthogonality between components to maximize variance, which is well suited to revealing the global structure of a data set but reduces the interpretability of biologically relevant pleiotropic signals (9). Conversely, NMF assumes strict non-negativity of component weights, which prevents it from describing component activation or inhibition relative to the reference condition used to normalize a transcriptomic compendium (10).

ICA treats the transcriptome as a summation of independent regulatory signals, each of which represents a subset of the TRN corresponding to an independently modulated set of genes (1). ICA decomposes the gene expression compendium (**X** matrix) into two matrices, one representing the individual source signals (iModulons; columns in the **M** matrix), and the other representing the strength of those signals across conditions (activities, in the **A** matrix), following the relationship **X** = **M** × **A** (Fig. 1c).

To conceptually distinguish ICA-derived components from traditional bottom-up regulons, Sastry et al., coined the term “iModulon” to describe top-down regulatory networks extracted from transcriptome data sets (1). The outcome of this ICA decomposition can vary between runs because it addresses a non-convex optimization problem (i.e., maximizing statistical independence) (11). Therefore, assessing the reproducibility of the decomposition is a key step in the iModulon workflow.

In practice, the workflow runs FastICA multiple times from different random seeds, with 100 iterations as the default setting in the current iModulon workflow (12). This iteration count reflects an empirical sampling depth chosen to reduce dependence on any single random initialization of the ICA optimization. For example, the original *E. coli* PRECISE study used 256 FastICA starts and retained components that clustered reproducibly (1). The resulting independent components are then compared across runs through DBSCAN clustering, where components that consistently appear across runs are retained as “robust components” (13). This ensures that the final iModulons reflect coherent regulatory signals that remain conserved even across disparate data sets. For instance, a CysB iModulon was recovered with 87% overlap across five independent *E. coli* data sets (13). Likewise, ICA applied to *Bacillus subtilis* transcriptome compendia has found that many iModulons—especially those of global regulators—decomposed from disparate datatypes, such as microarray and RNA-Seq transcriptomes, remained conserved (12). This conservation arises because genes are grouped into signals purely on the basis of expression patterns. Thus, regulatory modules that coherently affect gene expression across conditions emerge whenever the data span those conditions. Once the iModulons are computed, information on various molecular mechanisms is used to knowledge-enrich the regulatory signal. As shown in iModulonDB, most iModulons are biologically interpretable signals that complement previous information about regulons (14, 15).

A critical step in the iModulon workflow is selecting the optimal dimensionality of **X**, which defines the total number of components the algorithm attempts to extract from the data set. To prevent the artificial mixing of signals (under-decomposition) or the fragmentation of intact modules (over-decomposition), the OptICA method is used to automatically identify the optimal dimensionality, defined as the largest dimensionality that yields few single-gene components (16). Importantly, although known regulons were used in the original OptICA study to benchmark dimensionality choices, curated TRN information is not required a priori for the OptICA procedure. This feature enables its application to non-model organisms for which prior regulatory knowledge is limited, as discussed in subsequent paragraphs.

A key feature of ICA-mediated transcriptome decomposition is that each source signal is represented as a linear vector whose length equals the number of genes. This inclusion of all genes in each component provides a unique advantage over conventional methods, such as weighted gene co-expression network analysis (WGCNA), which enforce mutually exclusive gene grouping (17). In the ICA workflow, a single gene may belong to multiple iModulons, reflecting the interconnection between functionally relevant modules (18).

To translate these continuous vectors into discrete gene sets, weight thresholds must be applied. In this framework, the **M** matrix contains gene weights for each regulatory signal, where each value defines a gene’s dependence on that signal (Fig. 1c). Genes with weight values far from zero are strongly influenced by the signal, whereas genes with weights near zero are not. To define iModulons, the standard unsupervised approach relies on the D’Agostino *K*^2^ test to iteratively remove high-weight outlier genes until the remaining distributions become normal. When established TRNs are available, the normality test is calibrated to maximize F1 score between extracted components and known regulons (1). For understudied organisms lacking such TRN knowledge, unsupervised approaches, such as k-means clustering, can avoid curated biases and recover biologically meaningful modules (19). As a result, genes are classified into three clusters, and those in the two non-zero-centered clusters are grouped into the component (Fig. 1d). Overall, this integrated ICA-to-biology framework provides a robust method for dissecting transcriptional regulation and quantitatively tracking network activity in the era of large-scale omics data.

## THE SOFTWARE UNDERLYING THE iMODULON FRAMEWORK

The iModulon framework consists of standardized, user-friendly computational workflows. The Palsson Lab has developed iModulonMiner (https://github.com/SBRG/iModulonMiner), a workflow for iModulon computation and characterization, along with PyModulon (https://github.com/sbrg/pymodulon), a Python package for downstream iModulon analysis—together streamlining the application of ICA to new organisms and offering extensive analytical capabilities (1).

iModulonMiner provides a robust framework for collecting and processing publicly available RNA-seq data to perform ICA for the target organism. The iModulonMiner pipeline consists of five major steps, including (i) RNA-seq metadata mining from the NCBI SRA, (ii) raw data processing, (iii) quality control, (iv) ICA execution, and (v) iModulon characterization (Fig. 1f).

In step one, metadata for all RNA-seq data sets deposited in public repositories for the target organism are automatically retrieved. Users may also manually add metadata for external data sets, including user-generated data sets.

In step two, the raw fastq files corresponding to the selected RNA-seq data sets are downloaded using prefetch and fasterq-dump from the SRA Toolkit (2). The raw reads are then subjected to trimming, quality control using FastQC (http://www.bioinformatics.babraham.ac.uk/projects/fastqc/), alignment to the reference genome using Bowtie (20), strand direction curation using RSeQC (21), and gene expression calculation into log-transformed transcript-per-million (TPM). This step-wise workflow is implemented in Nextflow, streamlining user handling and enabling scalable execution (22). Quality-control metrics are summarized using MultiQC (22, 23), and a TPM-normalized gene expression matrix is generated as the final output of this process.

The third step involves not only filtering out low-quality sequencing data sets but also curating gene expression quality. Samples lacking biological replicates or showing poor correlation between biological replicates are excluded to ensure data fidelity (1). In this step, metadata can be further revised by the user to include additional experimental parameters, assigning biological features responsible for the observed transcriptional changes—information that is important for downstream iModulon annotations. In addition, the log TPM-normalized gene expression profiles can be further normalized against reference conditions to generate a gene expression fold-change matrix (12). Also in this step, reference conditions can be assigned independently for each project to minimize experimental batch effects and ensure that the observed transcriptional differences are attributable mainly to biological factors rather than protocol-specific biases. However, this strategy comes at the cost of the ability to compare gene expression changes across different projects.

Using the reference-normalized gene expression compendium, the fourth step performs ICA to extract independent biological signals. As described above, ICA is executed multiple times with different random seeds to identify robust components, while optimal dimensionality is selected to avoid over-decomposition.

Finally, in step five, individual iModulons are annotated using the PyModulon package, with user-guided interactive curation. Characterizing iModulons includes analyzing enriched gene functions within each iModulon, associating biological attributes with iModulon activities, and comparing the results with known regulatory information. Notably, because iModulons likely represent regulation mediated by one or a few transcriptional regulators, comparison with known regulons helps determine both the thresholds for iModulon gene membership and the interpretation of iModulon functions. Specifically, genes with high weights in an iModulon may overlap significantly with known regulons, enabling the iModulon to be assigned as a regulatory module governed by the corresponding regulator. At the same time, users may manually refine the weight thresholds to better represent the underlying regulatory architecture.

Although the pipeline incorporates computationally intensive machine-learning analyzes of large transcriptomic data sets—consisting of thousands of genes across hundreds to thousands of conditions—iModulonMiner is designed for scalability. For example, RNA-seq data processing (step two) is parallelized through the Nextflow implementation and takes only a few hours to handle hundreds of samples (12). The most resource-intensive step is ICA execution (step four); however, it remains tractable on modern hardware. In one benchmark, 100-cycle iterated ICA of the *E. coli* PRECISE-1K compendium (1,035 samples × 4,257 genes, approximately 80 MB gene expression matrix) at 200 dimensions was completed in under 8 min on a 64-core AMD Ryzen Threadripper workstation with 256 GB RAM (12). Subsequent DBSCAN clustering took about 1.1 min, and final matrix processing took about 16 s. This run produced approximately 2.2 GB of temporary files, while the final M and A matrices were only 13.3 and 2.9 MB, respectively. These benchmark results demonstrate that the iModulonMiner pipeline can analyze large transcriptomic compendia on modern workstations within a reasonable timeframe.

In addition to annotating iModulons, the PyModulon package provides extensive analytical capabilities based on iModulon structures, including visualization of iModulon information. PyModulon also enables the interpretation of new RNA-seq samples using existing iModulon information. A common challenge in analyzing large-scale data is that newly generated data sets are typically not included in previous analyzes, often requiring researchers to rerun computationally intensive procedures just to incorporate a small amount of additional data. However, because ICA represents gene expression as a combination of previously learned source signals, new data can still be interpreted using existing results. In this framework, iModulons serve as the modular units of gene expression regulation, and each transcriptome can be described by a specific pattern of iModulon activities. Thus, iModulon activities for newly generated data sets can be inferred using previously derived modules (Fig. 1e). This capability allows researchers to (i) estimate iModulon activity in new transcriptomes, (ii) evaluate how well existing iModulons explain new data, and (iii) decide whether re-running ICA with additional data is necessary to capture novel regulatory signals. Together, these two Python packages make the iModulon framework easier to apply to new organisms, with streamlined input requirements.

## COMPUTATIONAL DESIGN PRINCIPLES AND LIMITATIONS

While top-down transcriptomic decomposition offers a scalable framework for expanding prokaryote TRNs, it is not without caveats. Since ICA is subject to run-to-run variability, repeated decompositions of the same expression matrix can yield somewhat different independent components depending on initialization and the dimensionality selected for analysis (5). Current iModulon workflows address this by performing many ICA runs with different random seeds and then clustering similar components, typically retaining only those that recur reproducibly across runs as “robust” signals. Even so, this may not guarantee complete recovery of all biologically meaningful modules, nor fully exclude weakly resolved or artifactual components that lead to false positives in the ICA results (16).

**Fig 2:**
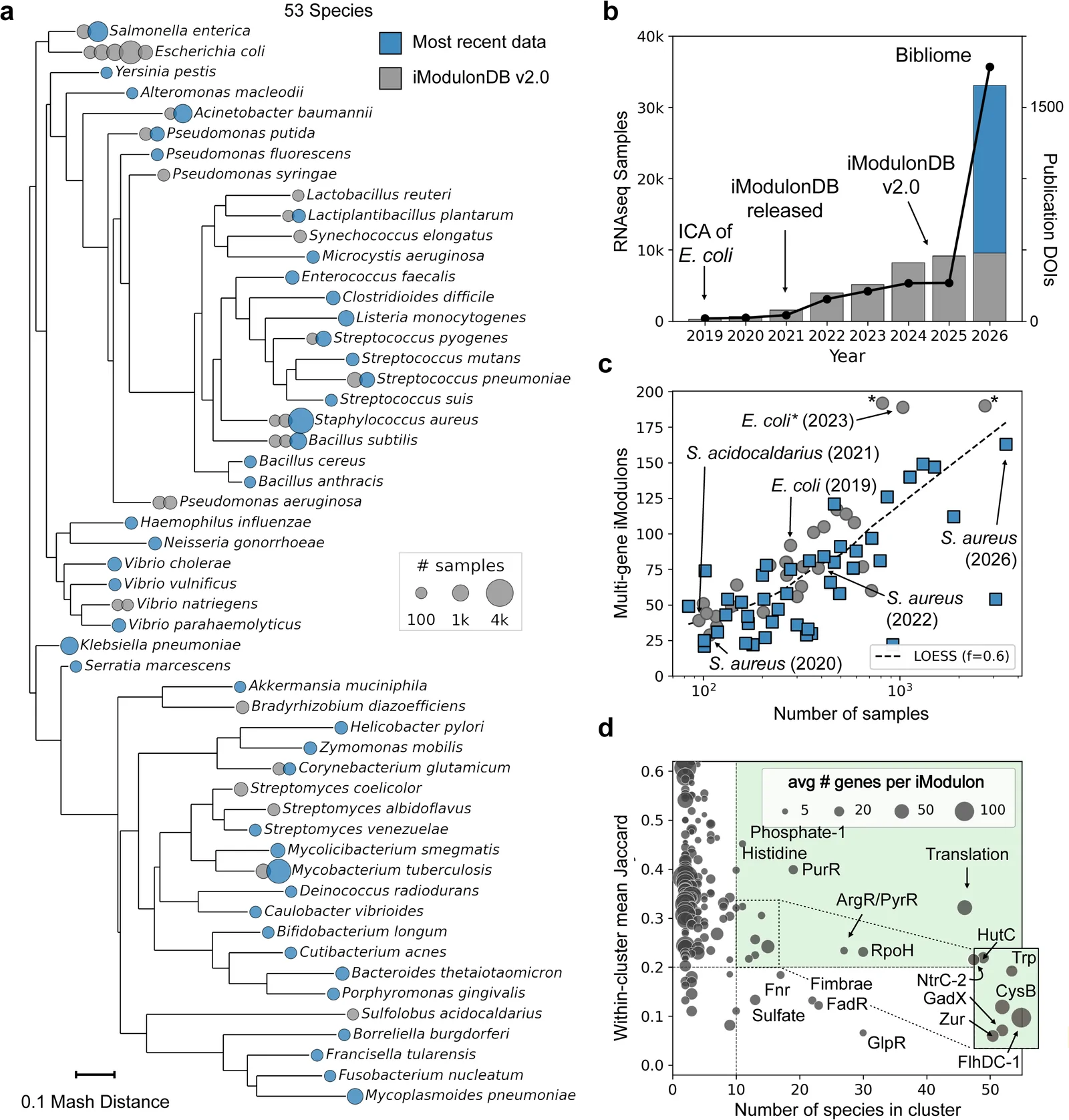
Scaling the iModulon framework across the prokaryotic phylogenetic tree. (**a**) Phylogenetic distribution of the 53 species in iModulonDB. Tree based on Mash distances (scale bar 0.1). Circle size at each tip indicates sample count; color indicates source (blue, this work; gray, previously published). Each circle represents an independent compendium. (**b**) Cumulative RNA-seq samples in iModulonDB (bars) and source publications contributing those samples (line, right axis), 2019–2026. Bars are stacked by source (gray: previously published; blue: this work). arrows mark major framework milestones. (**c**) Multi-gene iModulons recovered vs compendium size across 71 ICA decompositions. The dashed line shows LOESS smoother (frac = 0.6). Labels show within-organism scaling for *S. aureus* and *E. coli*, and the archaeal species *Sulfolobus acidocaldarius;* asterisks mark additional *E. coli* decompositions. (**d**) Cross-species iModulon conservation. Each point is a cluster of iModulons grouped by Jaccard similarity on a combined eggNOG/Pfam feature space (5,156 multi-gene iModulons clustered into 2,816 clusters; the 191 clusters spanning ≥2 species are shown). *x*-axis: number of species in the cluster; *y*-axis: mean within-cluster Jaccard; point size: average number of genes per iModulon in the cluster. shaded region marks clusters meeting both species-coverage (≥10) and similarity (≥0.20) thresholds, defining a shared regulatory toolkit across distantly related microbes.

In practice, the most effective safeguard against artifacts, and the primary driver of biological resolution, is the rigorous design and expansion of the underlying transcriptomic compendium. In ICA, regulatory signals can only be resolved when they vary sufficiently across the data set. Latent modules associated with rare stresses, alternative nutrient states, host-associated conditions, or strain-specific adaptations remain invisible in narrowly sampled data sets. As condition space broadens, these previously masked signals become statistically separable, improving both the resolution and biological interpretability of the resulting iModulons (18, 24). As a practical guide, one of the smallest iModulonDB compendia—on the order of 100 samples spanning ~50 distinct conditions—still recovers roughly 30 multi-gene iModulons (25), with additional modules emerging as the compendium expands (26) (Fig. 2c).

However, increasing compendium size must be carefully balanced against genetic consistency, as compendia containing genetically perturbed strains can introduce spurious changes in the gene weights in an iModulon. The initial attempt to map the *E. coli* TRN utilized the PRECISE data set (278 samples), which successfully mapped core metabolism but included two distinct K-12 strains, MG1655 and BW25113, yielding a specific “BW25113 iModulon” driven by transcriptomic discrepancies between the lineages (1). This compounding effect is magnified in larger data sets like PRECISE-1K, which incorporated targeted gene knockouts and adaptive laboratory evolution (ALE) endpoints. ICA applied to this heterogeneous data set effectively averaged across rewired regulatory states, yielding less clean iModulons with spurious gene memberships that reflect transient, strain-specific adaptations rather than shared native regulatory architecture (27). In contrast, reconstruction using the PRECISE-MG1655 data set—comprising 584 RNA-seq samples from a strictly wild-type background—yielded “cleaner” modules, where the prevalence of genes assigned to multiple iModulons was markedly reduced, with 82% of genes mapping uniquely to a single, well-defined iModulon (18). Because strain- and perturbation-driven signals can form their own iModulons, strain background matters when interpreting a decomposition: perturbed genes can be excluded from regulator–iModulon correlations, but telling native regulatory modules apart from strain- or perturbation-driven ones ultimately depends on curation (see “iModulonDB as community infrastructure” below).

A separate class of limitations concerns metadata rather than the decomposition itself. ICA operates on the expression matrix alone and is agnostic to sample annotation when computing the iModulons. Once computed, however, interpretation of the resulting iModulons depends on accurate, structured metadata describing each sample’s condition, strain, and experimental context.

Public repositories such as the SRA and BioProject typically provide this metadata as sparse, free-text fields that vary in completeness across submitters, with originating publications often not directly linked to the corresponding accession. Recovering this context—grouping samples into human-readable conditions, identifying genetic perturbations so they can be excluded from regulator–iModulon correlations, and tracing samples back to their source DOIs when not directly annotated—has historically required substantial manual curation. For the present work, new contributions were validated at submission through the upload pipeline introduced in iModulonDB 3.0, which we describe in subsequent sections. Sustained growth, however, will require pipelines that move beyond validation toward direct SRA integration and automated annotation of free-text experimental descriptions.

In addition to metadata, iModulons are annotated based on regulon, biochemical, genetic, and structural information available for the genes that comprise each iModulon. A fully annotated iModulon typically represents a recognizable cellular function. Thus, iModulons add an interpretable and understandable layer between the genotype and phenotype.

## iModulonDB AS COMMUNITY INFRASTRUCTURE

The growing scale of microbial transcriptomics demands not only analytical tools but also persistent, accessible infrastructure to host the resulting decompositions and make them reusable across studies. The Palsson Lab established iModulonDB (imodulondb.org) in 2021 as a centralized, interactive knowledgebase of curated iModulon sets derived from high-quality prokaryotic transcriptomic compendia (14). What began as a static repository for three model organisms has evolved into a platform for the curation and analysis of prokaryotic transcriptomes that now enables expansion across the microbial phylogenetic tree.

As a community resource, iModulonDB is organized hierarchically around three core entities—datasets (organism-specific compendia and their ICA decompositions), iModulons, and genes—each with interactive pages cross-linked to support both top-down browsing and gene-centric queries. Each data set comprises the matrices generated by the iModulon workflow (see “The Software Underlying the iModulon Framework” above), together with sample metadata linking each expression profile to its SRA accession and study of origin, and an organism-specific TRN reference where available (e.g., RegulonDB for *E. coli*; 8). This structured representation makes ICA decompositions persistent, citable, and machine-readable—addressing a major friction point in cross-study comparison.

The platform’s three separate releases trace a conceptual arc from passive repository to active workflow. iModulonDB 1.0 established the core dashboard architecture, with interactive iModulon and gene pages displaying gene-weight histograms, activity bar graphs, and regulator–activity scatter plots (14). iModulonDB 2.0 added project-level metadata and dataset-wide regulator screens (15), enabling integrative analyzes such as the *E. coli* fear–greed trade-off (27). iModulonDB 3.0 marks the most consequential shift: curation itself has moved onto the platform. Earlier releases required contributors to arrive with fully curated decompositions produced offline in PyModulon, restricting contributions to computationally fluent users. The current release introduces a private workspace in which contributors upload standard iModulonMiner outputs through a validation step that flags missing or inconsistent sample metadata, then iteratively refine annotations against rendered visualizations. This curation work—assigning iModulon names, surfacing candidate regulator associations from automated correlation screens, and flagging genomic or noise modules—runs against pre-computed dashboards rather than raw matrices, and the curated decomposition is released publicly with a single action. Strain information is part of this process: each data set page lists the strains in its compendium, and perturbed samples like gene knockouts tend to stand out in the activity and expression plots, helping curators spot and label strain- or perturbation-driven modules. Since this process relies on curation, some strain signals can slip through, so users should interpret iModulons with the strain metadata in mind.

Three features deserve specific mention from a data-science perspective. First, dashboards now embed publication abstracts and experimental variables directly within activity plots, with condition highlighting linked across charts so that selecting a condition updates all relevant views simultaneously. This connects each decomposition directly to its biological context, supporting interpretive work that previously required switching between the platform and primary literature.

Second, an integrated AI assistant has access to the current dashboard state and can pair textual responses with visualization actions—a query about which conditions most strongly activate a given iModulon, for example, can highlight the top differentially active samples on the activity bar chart. This provides a natural-language interface to the dashboards, with the underlying data displayed directly in the visualization for quick verification.

Third, each iModulon page cross-links to external structural and interaction databases—UniProt for gene-level annotation (28), STRING for protein–protein interactions (29), AlphaFoldDB for structures (30), and QSProteome for multi-protein complexes (25)—allowing co-modulation evidence from ICA to be checked against independent physical-association data.

Beyond curation, iModulonDB also makes each decomposition reusable. Every **M**-matrix in the database is stored in a machine-readable format with linked sample metadata, which lets downstream tools—notably PyModulon (see “The Software Underlying the iModulon Framework” above, Fig. 1e)—project new RNA-seq samples onto existing iModulons and interpret them in terms of pre-computed regulatory modules without re-running ICA. The combination of on-platform curation and reusable outputs has shifted iModulonDB from a destination for browsing pre-curated decompositions into a working infrastructure layer—a shift that has made community-driven expansion to dozens of new organisms tractable for experimental biologists. With the establishment of iModulonDB 3.0, the microbial community now has a deeply curated, accessible knowledge base that allows discovery, understanding, and design. This resource can thus grow over time and enable a series of new scientific inquiries, as we foreshadow in the next section.

## SCALING iMODULONS ACROSS THE MICROBIAL TREE

### Scaling single strain data sets

The combination of streamlined data analytic workflows (see “The Software Underlying the iModulon Framework” above) and on-platform curation (Section 5) has enabled a step change in the diversity of organisms represented in the iModulon framework. From the 15 species and 22 data sets of iModulonDB 2.0 (15), the resource now hosts ICA decompositions for 53 prokaryotic species and 71 data sets (Fig. 2a), with 43 new decompositions disclosed in the present work alongside 28 previously published. The underlying transcriptomic substrate spans 33,062 RNA-seq samples (Fig. 2b)—from model organisms and laboratory chassis to clinical pathogens, environmental isolates, and host-associated commensals. Of those samples, 23,500—over 70% of the cumulative total—were added in 2026 alone as part of the present expansion. Collectively, these samples span over 1,700 source publications, reflecting the breadth of the experimental literature now organized within the iModulon framework. This expansion was carried out primarily through a community curation pipeline coordinated alongside graduate coursework, supplemented by external published compendia integrated through the iModulonDB 3.0 upload portal.

With 71 data set decompositions available, we can examine how data set size relates to the number of regulatory modules ICA can identify. Across the 71 decompositions, the number of multi-gene iModulons recovered grows sublinearly with sample number (Fig. 2c, LOESS smoother). There are diminishing returns in the number of iModulons found as compendium size for a strain increases: a 400-sample compendium recovers ~80 multi-gene iModulons, and a 1,000-sample compendium recovers about 120. Sample number is a proxy for condition diversity because larger compendia tend to span a broader range of experimental conditions, and prior work indicates that condition diversity is a key driver of module recovery (17, 23); the 2023 *E. coli* PRECISE-1K decomposition, which recovers more modules than its sample count predicts, illustrates this (24). Because condition annotation is itself a curation-dependent quantity, the relationship between sample number and condition diversity is imperfect, accounting for the substantial scatter across the 71 compendia.

This scaling effect is recapitulated within individual organisms. The *S. aureus* trajectory spans over an order of magnitude in sample number, from 108 samples/29 iModulons (26) to 385/76 (31) to 3,467/163 (this work). *B. subtilis* traces a similar trajectory within RNA-seq compendia, from 265 samples/72 iModulons (32) to 782/142 (24). The *E. coli* trajectory shows the same pattern, from 278 samples/92 iModulons (1) to 1,035/189 in PRECISE-1K (27), where unusually broad condition diversity yields more modules than the cross-organism trend predicts at that sample count.

Beyond raw iModulon count, the gains from condition diversity are quantitatively measurable against known TRNs. The *B. subtilis* compendium expansion noted above raised median regulon recall against the SubtiWiki TRN from 0.53 to 0.67 while maintaining precision at 0.83, with 53 newly identified iModulons (24). Comparable TRN-coverage gains have been reported for *E. coli*, where the PRECISE-1K iModulons collectively account for 86% of all known strong-evidence regulatory interactions in RegulonDB (27). In both organisms, the newly captured modules reflect gene expression variation from novel stress regimes, nutrient limitations, and industrially relevant growth environments underrepresented in earlier compendia.

### Scaling across the phylogenetic tree

The standardization that iModulonDB provides also enables a class of analyses that one or a few organisms cannot support: systematic comparison of iModulon content across many bacterial species. To examine whether shared regulatory architecture is recoverable across distantly related species, we mapped the gene members of all 5,156 multi-gene iModulons across the 71 decompositions to a common feature space defined by the union of their eggNOG orthologous groups (33) and Pfam protein-domain families (34). Because eggNOG orthology assignments are sparse across less-studied genomes (covering 42.6% of member genes) while Pfam domain annotations are denser (76.4%), combining both identifier types raised feature coverage to 81.8% of member genes, allowing iModulons from distantly related species to be compared on a shared vocabulary. We then clustered iModulons by Jaccard similarity on this feature set. After removing promiscuous protein-domain families (those present in >5% of iModulons—predominantly broad transporter and signal-transduction motifs) and applying an anti-chaining constraint requiring at least five shared features per edge, the input iModulons grouped into 2,816 clusters, of which 191 contained iModulons from at least two species and 13 from at least 10 (Fig. 2d).

The most universally recurrent cluster corresponds to the translation iModulon—recovered in 46 of 53 species at a mean within-cluster Jaccard similarity of 0.32. A second tier of well-conserved clusters captures regulatory modules whose biological correlates are recognizable across phyla: phosphate uptake (phosphate-1; 11 species, mean Jaccard 0.45), histidine biosynthesis (Histidine; 10 species, 0.40), purine biosynthesis (PurR; 19 species, 0.40), amino-acid and pyrimidine biosynthesis (ArgR/PyrR; 27 species, 0.23), the heat-shock response (RpoH; 30 species, 0.23), flagellar assembly (FlhDC-1; 15 species, 0.24), sulfur metabolism (CysB; 13 species, 0.26), tryptophan biosynthesis (Tryptophan; 14 species, 0.31), and additional clusters spanning histidine catabolism (HutC), nitrogen regulation (NtrC-2), the GadX acid-stress response, and zinc homeostasis (Zur). Together, 13 clusters satisfy both wide species coverage (≥10 species) and reproducible gene-set composition (mean Jaccard ≥ 0.20), defining a quantitative “shared regulatory toolkit” present across distantly related bacteria. A separate set of six clusters spans many organisms but with lower mean Jaccard (Fnr, Sulfate, Fimbriae, FadR, RbsR, GlpR). These groupings reflect incidental protein-domain overlap—for example, shared acyl-CoA dehydrogenase or PTS-EIIC domains—rather than coherent shared regulatory programs. They illustrate why the Jaccard cutoff of 0.20 is necessary to distinguish genuine cross-species regulons from broad functional super-families.

### iModulonDB allows new queries

This comparative analysis represents a new class of questions that could not be answered before 71 standardized decompositions existed in one place. What was previously possible only one organism at a time—characterizing a regulator’s iModulon, identifying its members—becomes possible across many species. A regulator-by-regulator literature comparison becomes a quantitative cross-species recurrence analysis. The pattern that emerges—a small core of universally conserved regulatory programs plus a long tail of lineage- and niche-specific modules—is the kind of finding that the iModulon framework was always implicitly making about individual organisms, now demonstrated at scale.

The 53 organisms span broad phylogenetic diversity across the bacterial tree (Fig. 2a), with substantial sampling depth within the Firmicutes and Gammaproteobacteria and broader, but shallower, coverage across the Actinobacteria, Bacteroidetes, and other lineages. The breadth was made tractable by the curation infrastructure described in see “The Software Underlying the iModulon Framework” above. Sustaining expansion at the pace of public RNA-seq deposition, however, will require curation pipelines that move beyond manual annotation—direct integration with the SRA, LLM-assisted curation of sample metadata, and end-to-end automation from raw deposit to curated decomposition. Whether that trajectory can be realized depends on the design principles addressed in Section 4.

## OUTLOOK

Today, new decompositions enter iModulonDB in two steps: first, the iModulonMiner pipeline (see “The Software Underlying the iModulon Framework” above) produces the decomposition, and, second, curators annotate it on the iModulonDB 3.0 workspace (Section 5). Closing this loop is the immediate engineering direction.

Direct ingestion of SRA data can replace manual compendium assembly, through queries specifying organism, sample type, and quality criteria, with the iModulonMiner pipeline running on scheduled batches of new depositions. Free-text experimental metadata can be parsed by LLMs into the structured condition annotations upon which iModulon interpretation depends, with confidence scores propagated to flag samples that require human review. AI assistants integrated into the iModulonDB dashboard already draft iModulon names and regulator associations from the underlying gene weights and activity patterns; the engineering step is to flow those suggestions directly into the annotation files curators submit, so curators validate rather than annotate from scratch.

iModulonDB currently presents iModulons one organism at a time and one data type at a time. Both dimensions can be extended within the existing infrastructure. The cross-species conservation analysis was performed offline against downloadable outputs (Fig. 2c); the next step is to make these comparisons available within iModulonDB itself, where cross-species iModulon membership becomes a route to functional annotation. Extending across data types is a parallel step: fModules from genome-wide fitness compendia (35) and piModulons from matched proteomes (36) produce the same gene-weight and activity-matrix outputs as transcriptomic iModulons, so the existing dashboards and curation workflow apply directly. In each case the analytical work is established; what remains is engineering.

In realizing the applications previewed at the outset, the iModulon framework has already enabled several distinct classes of biological discovery. Genes of unknown function can be placed into physiological context when they appear with annotated pathway genes or regulons within the same iModulon (31). The boundaries of biological units, such as biosynthetic gene clusters, stress-response systems, or polysaccharide utilization loci, can be refined by co-regulation rather than by genome proximity alone (37, 38). At a larger scale, iModulon activities serve as variables that describe the state of the cell, making it possible to follow regulatory programs through time (39), compare strains (26), or quantify global allocation strategies, such as growth versus stress readiness (1). More excitingly, these same properties make iModulons an emerging tool in synthetic biology: instead of modifying one gene at a time, entire regulatory programs can be transferred, amplified, or rebalanced to produce complex phenotypes (40, 41). Thus, iModulon analysis provides a practical bridge from transcriptomic data to biological discovery and, increasingly, to module-level design, making the framework accessible to a broader community of biologists and engineers.

These applications broaden as the resource grows. The iModulonDB 3.0 upload portal introduced in this work enabled rapid community curation, scaling the resource from 15 species and 22 data sets (15) to 53 species and 71 data sets (Fig. 2d). The cross-species conservation analysis presented here (Fig. 2c) demonstrates that systematic cross-species iModulon comparison is now tractable at the scale the resource supports. RNA-seq deposition rates continue to accelerate, and each new compendium added to iModulonDB compounds the framework’s value: more samples per organism resolve additional iModulons, broader phylogenetic coverage reveals more universally conserved regulatory programs, and denser cross-links to UniProt, STRING, AlphaFoldDB, QSProteome, and other annotation resources enrich the biological context surrounding each module. Automation of the curation pipeline and integration across species and modalities allow the framework to scale with the data rather than lag behind it. Methodological extensions for single-cell and spatial RNA-seq, metatranscriptomes, and host-pathogen co-cultures will open further classes of regulatory biology beyond the current bulk-population scope. Together, these developments position the iModulon framework—anchored by the iModulonDB resource—to grow into an increasingly comprehensive reference for prokaryotic gene regulation, expanding in detail and connectivity with each new deposited sample, organism, and data type.

## Supplementary Material

Reviewer comments

## SUPPLEMENTAL MATERIAL

OPEN PEER REVIEW reviewer-comments.pdf An accounting of the reviewer comments and feedback.

ASM does not own the copyrights to Supplemental Material that may be linked to, or accessed through, an article. The authors have granted ASM a non-exclusive, world-wide license to publish the Supplemental Material files. Please contact the corresponding author directly for reuse.
