# Supplementary material for: The iModulon framework: how x-AI reveals microbial regulatory logic
Source: mSystems. 2026 Aug 12;11(9):e00228-26. doi: 10.1128/msystems.00228-26 (PMC13595982; doi:10.1128/msystems.00228-26)
Supplement: Supplementary file 1 [file reviewer-comments.pdf]

ASM | mSystems

The iModulon Framework: How x-AI reveals Microbial Regulatory Logic

Kangsan Kim, Edward Catiou, Yongjae Lee, Dukwon Lee, Chaewon Lee, Jiwon lee, Jongoh Shin, Bernhard Palsson, and Byung-Kwan Cho

Corresponding Author(s): Byung-Kwan Cho, Korea Advanced Institute of Science and Technology

Review Timeline:

Submission Date:	May 22, 2026
Editorial Decision:	June 24, 2026
Revision Received:	July 14, 2026
Accepted:	July 23, 2026

Editor: James Chappell

Reviewer(s): Disclosure of reviewer identity is with reference to reviewer comments included in decision letter(s). The following individuals involved in review of your submission have agreed to reveal their identity: Jason E McDermott (Reviewer #2)

Transaction Report:

(Note: With the exception of the correction of typographical or spelling errors that could be a source of ambiguity, letters and reports are not edited. The original formatting of letters and referee reports may not be reflected in this compilation.)

DOI: https://doi.org/10.1128/msystems.00228-26

Re: mSystems00228-26 (**The iModulon Framework: How x-AI reveals Microbial Regulatory Logic**)

Dear Prof. Byung-Kwan Cho:

Thank you for the privilege of reviewing your work. Below you will find my comments, instructions from the mSystems editorial office, and the reviewer comments.

Please return the manuscript within 60 days; if you cannot complete the modification within this time period, please contact me. If you do not wish to modify the manuscript and prefer to submit it to another journal, notify me immediately so that the manuscript may be formally withdrawn from consideration by mSystems.

Revision Guidelines
To submit your modified manuscript, log into the submission site at https://msystems.msubmit.net/cgi-bin/main.plex. Go to Author Tasks and click the appropriate manuscript title to begin. The information you entered when you first submitted the paper will be displayed; update this as necessary. Note the following requirements:

• Upload point-by-point responses to the issues raised by the reviewers in a file named "Response to Reviewers," NOT in your cover letter.
• Upload a compare copy of the manuscript (without figures) as a "Marked-Up Manuscript" file.
• Upload a clean .DOC/.DOCX version of the revised manuscript and remove the previous version.
• Each figure must be uploaded as a separate, editable, high-resolution file (TIFF or EPS preferred), and any multipanel figures must be assembled into one file.
• Any supplemental material intended for posting by ASM should be uploaded with their legends separate from the main manuscript. You can combine all supplemental material into one file (preferred) or split it into a maximum of 10 files with all associated legends included.

For complete guidelines on revision requirements, see our Submission and Review Process webpage. Submission of a paper that does not conform to guidelines may delay acceptance of your manuscript.

Data availability: ASM policy requires that data be available to the public upon online posting of the article, so please verify all links to sequence records, if present, and make sure that each number retrieves the full record of the data. If a new accession number is not linked or a link is broken, provide mSystems production staff with the correct URL for the record. If the accession numbers for new data are not publicly accessible before the expected online posting of the article, publication may be delayed; please contact production staff (mSystems@asmusa.org) immediately with the expected release date.

Publication Fees: For information on publication fees and which article types are subject to charges, visit our website. If your manuscript is accepted for publication and any fees apply, you will be contacted separately about payment during the production process; please follow the instructions in that e-mail. Arrangements for payment must be made before your article is published.

ASM Membership: Corresponding authors may join or renew ASM membership to obtain discounts on publication fees. Need to upgrade your membership level? Please contact Customer Service at Service@asmusa.org.

The ASM Journals program strives for constant improvement in our submission and publication process. Please tell us how we can improve your experience by taking this quick Author Survey.

Thank you for submitting your paper to mSystems.

Sincerely,
James Chappell
Editor
mSystems

Reviewer #1 (Comments for the Author):

Kim et al. present a manuscript named "The iModulon Framework: How x-AI 1 reveals Microbial Regulatory Logic' as an Invited Minireview, which describes the iModulon framework and associated database that they have been developing in recent years. The manuscript is well written and provides a comprehensive overview of their recent work on this tool. I only have a few minor suggestions.
Minor comments:

1. Line 100: "In practice, the workflow runs FastICA multiple times (e.g., 100 iterations)", How does one choose how many times is needed ? Currently it sounds arbitrary, a half sentence stating what is typically needed and why could clarify that.
2. Line 121: "select the optimal dimensionality by balancing the recovery of known regulators against ...": this sounds like it needs previous knowledge of TRN to determine the number of components. My understanding is that this is not the case, so this could be clarified. If it is the case this is a major limitation which would deserve further discussion.
3. Line 141-144: twice "for understudied organisms lacking ..."
4. Line 253: "As condition space broadens". It could be beneficial for readers to get a sense of how many conditions are even needed to see anything. In case of labs wanting to apply this to their own datasets, which might not always span many conditions.
5. Line 256: "Genetic consistency". Since the authors report such a large increase in number of datasets and species covered with the new release of their database with now 71 decompositions, how do they deal with the genetic strain diversity ? Should users expect to take that into account when looking at iModulons in the database and keep in mind that some can be due to differences in genetic backgrounds ? Or did they typically only group samples together by strain ?
6. Generally, the manuscript is quite technical and would likely go over the heads of biologists with no computational/systems biology background. If the authors want to also reach that demographic, I think it could be beneficial to take the hand of the reader a little bit more on topics including what types of question can someone explore with such tools, what type of biological discovery can one do, why would one want to identify such groups of genes etc... One place where I could see that being added is in the introduction, which is currently very short and only about data.

Reviewer #2 (Comments for the Author):

The paper by Kim et al. describes the iModulon framework, provides a review of the principles and approaches for determining regulatory structure from transcriptomics datasets, and gives an overview of the latest update to the database and resource. The paper is a good overview of an important bacterial resource. I have the following comments and suggestions to improve the paper:
1. The number of species (53) versus ICA decompositions (71) is a bit confusing in the Abstract. I believe from the rest of the paper that this is because some of the species have multiple datasets and so ICA was applied to each? Some context here would be helpful to the reader.
2. Ending the Abstract with a sentence about 'engineering directions' is not strong - especially for mSystems. A sentence summarizing the impact of the tool/database would be better here.
3. Line 115: the statement should have a citation or some evidence to support it.
4. Line 162: 'reposited' is a typo?
5. Line 165: Step two is confusing it sounds like step 1 is retrieving all the data, then step 2 is retrieving all the data. Can you please clarify what the difference is between these two steps?
6. Line 296: 'quarriable' is a typo
7. Line 301: 'phylons' requires a definition
8. Lines 384-385: the statement about condition diversity driving discovery of additional regulatory modules needs support from data or a citation.
9. Line 409: The statement that the 71 decompositions map to a common feature space needs some clarification. The citations for this are to eggNOG and Pfam - which could be used for this purpose, but likely not both at the same time?
10. Line 671: 'iMoculon' is a typo

Point-by-point response to reviewer comments

The iModulon Framework: How x-AI reveals Microbial Regulatory Logic (mSystems00228-26)

Reviewer #1 (Comments for the Author):

Kim et al. present a manuscript named "The iModulon Framework: How x-AI 1 reveals Microbial Regulatory Logic' as an Invited Minireview, which describes the iModulon framework and associated database that they have been developing in recent years. The manuscript is well written and provides a comprehensive overview of their recent work on this tool. I only have a few minor suggestions.

Minor comments:

1.Line 100: "In practice, the workflow runs FastICA multiple times (e.g., 100 iterations)", How does one choose how many times is needed ? Currently it sounds arbitrary, a half sentence stating what is typically needed and why could clarify that.

Response: We thank the reviewer for the comment. We revised the text to clarify that this number is an empirical reproducibility parameter used to sample solutions of the non-convex ICA optimization. Published iModulon studies have used repeated FastICA runs followed by DBSCAN clustering to retain components that recur across independent random initializations. The original *E. coli* PRECISE study used 256 random starts (PMID: 31797920), whereas a subsequent cross-dataset analysis, and the established pymodulon workflow use 100 random starts (PMID: 33529205; 39441835). Thus, the essential criterion is not the exact number of starts, but the reproducible recovery of components across repeated decompositions.

Lines 113-118: In practice, the workflow runs FastICA multiple times (e.g., 100 iterations), starting from different random seeds, typically using 100 iterations as the default setting in current iModulon workflow (Sastry et al. 2024). This iteration is an empirical sampling depth used to reduce dependence on any single random initialization of the ICA optimization. For example, the original E. coli PRECISE study used 256 FastICA starts and retained components that clustered reproducibly (1).

2.Line 121: "select the optimal dimensionality by balancing the recovery of known regulators against ...": this sounds like it needs previous knowledge of TRN to determine the number of components. My understanding is that this is not the case, so this could be clarified. If it is the case this is a major limitation which would deserve further discussion.

Response: We agree that the original wording was misleading. Known regulons were used in the original development and benchmarking of OptICA to evaluate under-decomposition, but the operational criterion used by OptICA is an unsupervised signal of over-decomposition: the emergence of single-gene components. We have revised

the sentence to clarify that OptICA can be applied without a curated TRN, while known regulons remain useful for downstream annotation and validation when available.

Lines 135-142: To prevent the artificial mixing of signals (under-decomposition) or fragmentation of intact modules (over-decomposition), the OptICA method is utilized to automatically select the optimal dimensionality, defined as the largest dimensionality that yields few single-gene components (13). Importantly, although known regulons were used in the original OptICA study to benchmark dimensionality choices, curated TRN information is not required a prior for the OptICA procedure. This feature enables its application to non-model organisms for which prior regulatory knowledge is limited, as discussed in subsequent paragraphs.

> 3.Line 141-144: twice "for understudied organisms lacking ..."

Response: We thank the reviewer for pointing this out. The redundant phrase was removed from the sentence.

Lines 158-160: For understudied organisms lacking such TRN knowledge, unsupervised approaches such as k-means clustering can avoid curated biases and recover biologically meaningful modules (19)~~.~~

> 4.Line 253: "As condition space broadens". It could be beneficial for readers to get a sense of how many conditions are even needed to see anything. In case of labs wanting to apply this to their own datasets, which might not always span many conditions.

Response:
We thank the reviewer for this practical suggestion. We have added a concrete guideline to the passage to give readers a reference point for the scale at which ICA becomes informative on their own data.

Lines 269-272: "…one of the smallest compendia in iModulonDB — on the order of 100 samples spanning ~50 distinct conditions — still recover roughly 30 multi-gene iModulons (29), with additional modules emerging as the compendium expands (30) (**Fig. 2c**)."

> 5.Line 256: "Genetic consistency". Since the authors report such a large increase in number of datasets and species covered with the new release of their database with now 71 decompositions, how do they deal with the genetic strain diversity ? Should users expect to take that into account when looking at iModulons in the database and keep in mind that some can be due to differences in genetic backgrounds ? Or did they typically only group samples together by strain ?

Response:
We thank the reviewer for this important point. Rather than restricting each decomposition to a single strain, we handle strain diversity through curation and make it visible to users. We have clarified this in two places. In the limitations section (Section 4), we now note that strain- and perturbation-driven signals can form their own iModulons, so strain background matters when interpreting a decomposition, and that separating these from native regulatory modules depends on curation.
Lines 288-292: Because strain- and perturbation-driven signals can form their own iModulons, strain background matters when interpreting a decomposition: perturbed genes can be excluded from regulator–iModulon correlations, but telling native regulatory modules apart from strain- or perturbation-driven ones ultimately depends on curation (Section 5).
In the iModulonDB section (Section 5), we now note that each dataset page lists the strains in its compendium, that perturbed samples such as gene knockouts tend to stand out in the activity and expression plots (helping curators identify and label strain- or perturbation-driven modules), and that — because this relies on curation — some strain signal can persist, so users should interpret iModulons with the strain metadata in mind. Yes, we agree users should take strain background into account: as the manuscript notes, iModulons can capture strain differences and genetic alterations in addition to regulatory signals.
Lines 348-353: Strain information is part of this process: each dataset page lists the strains in its compendium, and perturbed samples like gene knockouts tend to stand out in the activity and expression plots, helping curators spot and label strain- or perturbation-driven modules. Since this process relies on curation, some strain signals can slip through, so users should interpret iModulons with the strain metadata in mind.

6.Generally, the manuscript is quite technical and would likely go over the heads of biologists with no computational/systems biology background. If the authors want to also reach that demographic, I think it could be beneficial to take the hand of the reader a little bit more on topics including what types of question can someone explore with such tools, what type of biological discovery can one do, why would one want to identify such groups of genes etc... One place where I could see that being added is in the introduction, which is currently very short and only about data.

Response: We agree that the manuscript was weighted toward the technical aspects of the iModulon framework. To make the review more accessible to potential users, including biologists and bioengineers, we have added a brief paragraph that introduces how iModulons can be used in practice for biological discovery and engineering applications. This addition explains how iModulons help convert transcriptomic data into coordinated regulatory programs, enabling functional inference, comparison of cellular states, and emerging module-level design strategies.

Lines 57-67: For an experimental biologist, the practical appeal of this decomposition is that it converts a transcriptome from a list of thousands of individually varying genes into a smaller set of dozens of co-regulated modules, each corresponding to a recognizable

cellular function. This reframing allows one to ask module-level questions — which functions are switched on under an experimental condition, when a program is deployed, and how its activity tracks with phenotype — and offers a route to place genes of unknown function into physiological context, refine the boundaries of biological units, and compare regulatory states across strains and conditions. We return to these applications, including emerging uses in module-level strain design, in Section 7; here, we describe the Data Science framework that generates iModulon structures for prokaryotic transcriptomes.

Lines 511-526: In realizing the applications previewed at the outset, the iModulon framework has already enabled several distinct classes of biological discovery. Genes of unknown function can be placed into physiological context when they appear with annotated pathway genes or regulons within the same iModulon (31). The boundaries of biological units, such as biosynthetic gene clusters, stress-response systems, or nutrient-utilization loci, can be refined by co-regulation rather than by genome proximity alone (36). At a larger scale, iModulon activities serve as variables that describe the state of the cell, making it possible to follow regulatory programs through time (37), compare strains (29), or quantify global allocation strategies such as growth versus stress readiness (1). More excitingly, these same properties make iModulons an emerging tool in synthetic biology: instead of modifying one gene at a time, entire regulatory programs can be transferred, amplified, or rebalanced to produce complex phenotypes (38, 39). Thus, iModulon analysis provides a practical bridge from transcriptomic data to biological discovery and, increasingly, to module-level design, making the framework accessible to a broader community of biologists and engineers. These applications broaden as the resource grows.

Reviewer #2 (Comments for the Author):
The paper by Kim et al. describes the iModulon framework, provides a review of the principles and approaches for determining regulatory structure from transcriptomics datasets, and gives an overview of the latest update to the database and resource. The paper is a good overview of an important bacterial resource. I have the following comments and suggestions to improve the paper:

1.The number of species (53) versus ICA decompositions (71) is a bit confusing in the Abstract. I believe from the rest of the paper that this is because some of the species have multiple datasets and so ICA was applied to each? Some context here would be helpful to the reader.

Response: We thank the review of this suggestion. The reviewer is correct, multiple decompositions were conducted and/or previously published for several species. We have added the following phrases to the manuscript:

Lines 29-32: We present iModulonDB 3.0 that contains 53 species and 71 ICA decompositions across 33,062 RNA-seq samples, “with several well-sampled species

(e.g., *E. coli, B. subtilis, S. aureus, P. aeruginosa*) represented by more than one compendium."

Lines 715-716: (Caption, Figure 2A) Each circle represents an independent compendium.

2.Ending the Abstract with a sentence about 'engineering directions' is not strong - especially for mSystems. A sentence summarizing the impact of the tool/database would be better here.

Response: We thank the reviewer for this suggestion. We toned down on 'engineering directions' and revised the Abstract as below.

Lines 37-40: Together, these advances position the iModulon framework as an accessible, community-driven approach for reading accumulating public transcriptomes as reusable regulatory programs, enabling biological discovery and module-level design in synthetic biology.

3.Line 115: the statement should have a citation or some evidence to support it.

Response: We thank the reviewer for pointing this out. Relevant iModulon DB papers were cited in support of the statement.

Lines 130-132: As shown in iModulonDB, the majority of iModulons are biologically interpretive signals that complement previous information about Regulons (14, 15).

4.Line 162: 'reposited' is a typo?

Response: We thank the reviewer for their careful reading. We have changed the word to "deposited" (Line 178).

5.Line 165: Step two is confusing it sounds like step 1 is retrieving all the data, then step 2 is retrieving all the data. Can you please clarify what the difference is between these two steps?

Response: We thank the reviewer for pointing this out. Step one involves collecting metadata for RNA-seq datasets, whereas step two involves downloading and processing the corresponding raw sequencing data. We have revised the manuscript to clarify this distinction.

Lines 178-182: In step one, metadata for all RNA-seq datasets deposited in public repositories for the target organism are automatically retrieved. Users may also manually add metadata for external datasets, including user-generated datasets.

In step two, the raw fastq files corresponding to the selected RNA-seq datasets are downloaded using prefetch and fasterq-dump from the SRA Toolkit (2).

6.Line 296: 'quarriable' is a typo

Response: We thank the reviewer for their careful reading. We have changed the word to "accessible" (Line 317)

7.Line 301: 'phylons' requires a definition

Response: We thank the reviewer for their comment. We have changed the sentence to read "expansion across the microbial phylogenetic tree." (Lines 322-323)

8.Lines 384-385: the statement about condition diversity driving discovery of additional regulatory modules needs support from data or a citation.

Response: We thank the reviewer for their comment. We have revised the passage to support the claim with both citations and a supporting example. Specifically, we now cite prior work indicating that condition diversity drives module recovery (refs 17, 23) and point to the *E. coli* PRECISE-1K decomposition (ref 24), which recovers more modules than its sample count alone predicts, as an illustrative case. We have also softened the causal phrasing to reflect that sample number serves as a proxy for condition diversity rather than asserting the relationship directly. These changes appear on lines 385–389.

Lines 407-411: Sample number is a proxy for condition diversity because larger compendia tend to span a broader range of experimental conditions, and prior work indicates that condition diversity is a key driver of module recovery (17, 23); the 2023 *E. coli* PRECISE-1K decomposition, which recovers more modules than its sample count predicts, illustrates this (24).

9.Line 409: The statement that the 71 decompositions map to a common feature space needs some clarification. The citations for this are to eggNOG and Pfam - which could be used for this purpose, but likely not both at the same time?

Response:
We thank the reviewer for raising this point. The reviewer is correct that eggNOG (orthologous groups) and Pfam (protein domains) are distinct annotation types; we combined them deliberately, as a union of per-gene identifiers, for coverage. Across our 71 species, eggNOG assignments are sparse (42.6% of member genes) while Pfam is denser (76.4%), and taking the union raises coverage to 81.8%. eggNOG alone would leave over half of member genes without a feature and bias the comparison toward

well-studied model organisms — the opposite of what a phylogeny-spanning analysis requires. We have revised the passage to make this explicit.

Lines 435–441: "...we mapped the gene members of all 5,156 multi-gene iModulons across the 71 decompositions to a common feature space defined by the union of their eggNOG orthologous groups (32) and Pfam protein-domain families (33). Because eggNOG orthology assignments are sparse across less-studied genomes (covering 42.6% of member genes) while Pfam domain annotations are denser (76.4%), combining both identifier types raised feature coverage to 81.8% of member genes, allowing iModulons from distantly related species to be compared on a shared vocabulary."

10.Line 671: 'iMoculon' is a typo

Response: We thank the reviewer for their careful reading. We have changed the word to "iModulon" (Line 708).

Re: mSystems00228-26R1 (**The iModulon Framework: How x-AI reveals Microbial Regulatory Logic**)

Dear Prof. Byung-Kwan Cho:

Your manuscript has been accepted, and I am forwarding it to the ASM production staff for publication. Your paper will first be checked to make sure all elements meet the technical requirements. ASM staff will contact you if anything needs to be revised before copyediting and production can begin. Otherwise, you will be notified when your proofs are ready to be viewed.

Data Availability: ASM policy requires that data be available to the public upon online posting of the article, so please verify all links to sequence records, if present, and make sure that each number retrieves the full record of the data. If a new accession number is not linked or a link is broken, provide production staff with the correct URL for the record. If the accession numbers for new data are not publicly accessible before the expected online posting of the article, publication may be delayed; please contact ASM production staff immediately with the expected release date.

Publication Fees: For information on publication fees and which article types have charges, please visit our website. We have partnered with Copyright Clearance Center (CCC) to collect author charges. If fees apply to your paper, you will receive a message from no-reply@copyright.com with further instructions. For questions related to paying charges through RightsLink, please contact CCC at ASM_Support@copyright.com or toll free at +1-877-622-5543. CCC makes every attempt to respond to all emails within 24 hours.

ASM Membership: Corresponding authors may join or renew ASM membership to obtain discounts on publication fees. Need to upgrade your membership level? Please contact Customer Service at Service@asmusa.org.

Embargo Policy: A press release may be issued as soon as the manuscript is posted on the mSystems Latest Articles webpage. The corresponding author will receive an email with the subject line "ASM Journals Author Services Notification" when the article is available online.

Cover Image Submissions: If you would like to submit a potential Cover Image, please email a file and a short legend to msystems@asmusa.org. Please note that we can only consider images that (i) the authors created or own and (ii) have not been previously published. By submitting, you agree that the image can be used under the same terms as the published article. Image File requirements: TIF/EPS, 7.5 inches wide by 8.25 inches tall (at least 2,250 pixels wide by 2,475 pixels tall), minimum 300 dpi resolution (600 dpi preferred), RGB, and no figure elements, e.g., arrows or panel labels. The legend should be a short description of the image, 1-2 sentences recommended. Please download and use this interactive template in Adobe the https://journals.asm.org/pb-assets/html/asm-cover-sizing-tool.html to ensure that your proposed cover image meets our size requirements (https://journals.asm.org/pb-assets/pdf-text-excel-files/ASM-Interactive-Sizing-Cover-Template-1715689791.pdf).

Thank you for submitting your paper to mSystems.

Sincerely,
James Chappell
Editor
mSystems
